# Intranasal administration of dantrolene increased brain concentration and duration

**DOI:** 10.1371/journal.pone.0229156

**Published:** 2020-03-11

**Authors:** Jintao Wang, Yun Shi, Shuchun Yu, Yan Wang, Qingcheng Meng, Ge Liang, Maryellen F. Eckenhoff, Huafeng Wei

**Affiliations:** 1 Department of Anesthesiology and Critical Care, University of Pennsylvania Perelman School of Medicine, Philadelphia, PA, United States of America; 2 Department of Anesthesiology, Tongji Hospital, Tongji Medical College, Huazhong University of Science and Technology, Wuhan, China; 3 Department of Anesthesiology, Children’s Hospital of Fudan University, Shanghai, China; 4 Department of Anesthesiology, The Second Affiliated Hospital to Nanchang University, Nanchang, Jiangxi, China; 5 Department of Anesthesiology, Shandong Provincial Hospital Affiliated to Shandong University, Jinan, China; Imperial College London, UNITED KINGDOM

## Abstract

Dantrolene has been demonstrated to be neuroprotective for multiple neurodegenerative diseases. However, dantrolene’s limited penetration into the CNS hampers its effectiveness as a neuroprotective agent. Here, we studied whether the intranasal administration of dantrolene provided better penetration into the brain than the commonly used oral approach. C57BL/6 mice, aged 2–4 months, received a single dose of either intranasal or oral dantrolene (5mg/kg). Inhibition of dantrolene clearance from the brain was examined by co-administration with P-gp/BCRP inhibitors, nimodipine or elacridar. The concentration of dantrolene in the brain and plasma was measured at 10, 20, 30, 50, 70, 120, 150 and 180 minutes after administration. Separate cohorts of mice were given intranasal dantrolene (5mg/kg) or vehicle, 3 times/ week, for either 3 weeks or 4 months, to examine potential adverse side effects on olfaction and motor coordination, respectively.

We found that Dantrolene concentrations were sustained in the brain after intranasal administration for 180 min, while concentrations fell to zero at 120 min for oral administration. Chronic use of intranasal dantrolene did not impair olfaction or motor function in these mice. Blood brain barrier pump inhibitors did not further increase dantrolene peak concentrations in the brain. Our results suggested that Intranasal administration of dantrolene is an effective route to increase its concentration and duration in the brain compared to the oral approach, without any obvious side effects on olfaction or motor function.

## Introduction

Dantrolene, an antagonist of the ryanodine receptor (RYR) calcium (Ca^2+^) channel, which is located in the membrane of the sarcoplasmic reticulum (SR) in muscle cells and the endoplasmic (ER) reticulum in neurons, is clinically used to treat muscle spasticity and malignant hyperthermia (MH) in patients, reducing MH mortality from 64% to 1.4% [1, 2]. Dantrolene, in various animal models, has been shown to be neuroprotective in many neurodegenerative diseases, including cerebral ischemia [3, 4], Huntington’s disease [5], amyotrophic lateral sclerosis [6], trauma [7], and seizers [8]. Dantrolene has also been demonstrated to reduce mortality in an animal model of sepsis [9]. One early study of intraperitoneal injections of dantrolene in a familiar Alzheimer’s disease (FAD) animal model demonstrated improved neuropathology, but failed to examine cognition [10]. Recently, it has been demonstrated that both subcutaneous (SQ) and oral dantrolene have reduced amyloid pathology and memory loss in different Alzheimer disease (AD) animal models [11–13]. It seems that excessive Ca^2+^ release from the SR/ER plays an important role in inducing and/or aggravating cell stress and damage, leading to eventual muscle or neuronal damage. This could be ameliorated by dantrolene.

Although dantrolene is a promising treatment for neurodegenerative diseases in various animal models, a major obstacle is the limited penetration of dantrolene into the CNS. Dantrolene has two properties, though, working in its favor to penetrate the CNS. It is both lipid soluble and has a molecular weight of 314 g/mol. Lipid soluble drugs and drugs with molecular weights under 400 g/mol are expected to penetrate the blood brain barrier (BBB) readily. However, the ability of dantrolene to pass the BBB is still controversial with evidence for [14], and against [15] passage. The use of dantrolene for the treatment of AD or stroke would require chronic administration. Due to the limited penetration of dantrolene into the CNS from the blood, oral administration requires high doses of dantrolene to reach the therapeutic concentration threshold in the CNS, making patients prone to first pass liver metabolism and drug toxicity. Therefore, development of a method for elevating the dantrolene brain concentration for a longer duration is crucial to the future use of dantrolene as a treatment for Alzheimer’s and other neurodegenerative diseases. The intranasal route for drug delivery is an emerging, viable, and non-invasive means for treating CNS disorders. Intranasal drugs have been shown to rapidly enter the brain along both the olfactory and trigeminal nerves via both intracellular and extracellular routes [16–18]. Intranasal drug delivery, targeted to the CNS, has been shown to reduce systemic exposure and adverse systemic side effects [19].

In this study, we have demonstrated that intranasal administration of dantrolene in mice significantly increased the concentration and duration of dantrolene in the brain, compared to oral administration. This may provide a new approach to maximize the potential neuroprotective effects of dantrolene in various neurodegenerative diseases, while minimizing its toxicity and side effects.

## Materials and methods

### Animals

All animal procedures were approved by the Institutional Animal Care and Use Committee (IACUC) of the University of Pennsylvania. Male and female C57BL/6 mice (Charles River Laboratories, Inc. Wilmington, MA), 2–4 months old, weighing 25-35g, were used in all experiments. Mice were kept at 21–22°C with a 12-hour light-dark cycle with food and water ad libitum. All efforts were made to minimize pain and distress and the number of mice.

### Drug administration

For the pharmacokinetic studies, mice were randomly divided into two experimental groups; intranasal dantrolene for intranasal administration (n = 40) and oral dantrolene delivery (n = 30). The vehicle was the same formulation as RYANODEX(Eagle Pharmaceuticals, Inc.), consisting of 125mg mannitol, 25mg polysorbate 80, 4mg povidone K12 in 20 ml of ddH_2_O and pH adjusted to 10.3. Dantrolene (MilliporeSigma, St Louis, MO) was diluted in the vehicle to a concentration of 5mg/ml. For intranasal administration, the mice were held firmly in one hand and a total of 1 μl of drug formulation per gram of body weight was delivered using a pipette. Several key steps were required to assure accuracy of intranasal delivery: 1) the mouse’s head was parallel to the floor; 2) the mouse was not able to move the head or neck; 3) the ejected droplet was as small as possible; 4) 2–3 s was allowed between intranasal injections; 5) the mouse was held for 10–15 seconds after the delivery was finished. This procedure took about 10 min/ mouse. Oral administration was performed as previously described [20]. The mice were held as for the intranasal administration and 5μl of drug per gram of body weight was delivered using a gavage attached to a microliter syringe.

Inhibition of the BBB transport protein, P-glycoprotein breast cancer resistance (P-gp/BCRP), function has been shown to increase the brain/plasma concentration ratios of dantrolene (Fuchs et al, 2014). In an attempt to reduce dantrolene clearance from the brain, BBB pump inhibitors (nimodipine, elacridar) were given prior to intranasal dantrolene to a separate cohort of animals. Nimodipine and elacridar (Sigma, St Louis, MO) were diluted in the same vehicle as Ryanodex, described above, 2mg/ml and 10mg/ml respectively. A total of 1 μl of nimodipine or elacridar per gram of body weight was delivered by intranasal administration 30 min before intranasal administration of 5mg/ml dantrolene (1 μl/g of body weight). Dantrolene alone was used as the control. Blood and plasma dantrolene concentrations were examined 20 min after the intranasal administration of dantrolene.

For the drug safety studies, the potential adverse effects of chronic administration of dantrolene were examined. Separate cohorts of mice were randomly divided into groups which received intranasal dantrolene (5 mg/kg) or intranasal vehicle, 3 times/week, for either 3 weeks or 4 months, for behavioral studies as described below. There was no mortality in all groups.

### Sample collection and euthanasia

At the time of euthanasia, animals were anesthetized with 2–4% isoflurane and blood samples (0.2 ml) were obtained by cardiac puncture after 10, 20, 30, 50, 70, 120, 150 and 180 minutes of dantrolene administration. The animals were then euthanized by intracardiac perfusion and exsanguination with phosphate buffered saline to ensure that dantrolene was completely washed out of the cerebrovascular system before the brains were harvested. Heparin anticoagulated blood samples were centrifuged at 3000 rpm at 4°C for 10 minutes and the supernatant collected. Each brain was dissected and homogenized. All procedures were performed in the cold room (4°C). Both the plasma and brain samples were stored at -80°C and protected from light until assayed. Separate cohorts of mice were euthanized as above after 3 weeks or 4 months of chronic dantrolene administration for the olfaction and motor function tests, respectively.

### High performance liquid chromatography (HPLC)

The brain tissues for HPLC analysis were extracted as previously described [21]. Briefly, the frozen brain tissue was placed into 200 μl of mixture solution (acetonitrile: H_2_O, 2:1) and homogenized, the suspensions were then centrifuged at 4°C at 20,000 x g for 20 min. For the plasma samples, 200 μl of acetonitrile was added into the same volume of specimen solution and centrifuged. After the homogenization and centrifugation, 50 μl of supernatant was injected into HPLC for analysis.

An Agilent Hewlett Packard Model 1100 Series, high performance liquid chromatography (HPLC) system (Agilent Technologies, Wilmington, DE), equipped with a refractive index monitor, was used for quantitation of dantrolene concentrations in the blood and brain. Acetonitrile was used as component A of the mobile phase, and potassium phosphate buffer solution (pH 7.0) as component B. The mobile phase had a flow rate of 1.0 ml/min with a proportion 12% to 88% for components A and B of the mobile phase, respectively. Detection was performed with the UV detector at 254 nm.

### Behavioral assays for examination of adverse side effects

#### Buried food test

As this is the pilot study of administrating dantrolene via the intranasal approach and since chronic use will be needed for the treatment of Alzheimer’s or other neurodegenerative diseases, we further investigated whether the chronic use of intranasal dantrolene will impair the sense of smell. Olfaction was assessed in a separate cohort of mice after 3 weeks of intranasal dantrolene (5mg/kg, N = 10) or vehicle (equivalent volume, N = 10), using the buried food test[22]. Mice were randomly divided into two experimental groups (n = 10/group). Dantrolene or vehicle was administrated once a day, three times a week (every other day during weekdays). After 3 weeks of chronic administration, animals were subjected to the buried food test by an investigator who was blinded to the experimental groups. On day 1, cookies (1 cookie for 2 mice) were placed into the cages and left overnight. Cages were observed on the second day to make sure the cookies were consumed. On day 2 at about 4pm, food was removed from the cages and the testing mice were fasted overnight, water available. On day 3, at about 11am, mice were acclimated to the testing room for 1 hour. Mice were then individually placed into a clean cage with 3cm deep of bedding with a cookie buried 1cm beneath the bedding in one corner. The time it took the mouse to retrieve the food and hold it with the front paws was manually recorded, for a maximum of 900 seconds.

#### Rotarod test

As dantrolene has effects of muscle relaxant, we further investigated the effects of chronical use of dantrolene on motor function. Motor coordination was examined with a rotarod [23] in a separate cohort of mice that were given either intranasal dantrolene (5 mg/kg, N = 10) or vehicle (equivalent dose, N = 10), once a day, 3 times/ week, for 4 months. The animals received two 60s training trials on the rotarod (IITC Series 8, Life Sciences, Woodland Hills, CA) at 9 rpm with a 30 min interval between trials. The mice then underwent three test trials for a maximum of 120s at variable speed, 4–40 rpm, with a 60 min interval between trials. The time spent on the rotarod was recorded automatically for each mouse.

#### Statistical analysis

All data are reported as the Mean ± 95% CI and were analyzed by multiple t-tests using the Holm-Sidak method or by the nonparametric unpaired Mann-Whitney test (two-tailed), as described in each figure legend. The significance level for all of our analyses was set at 95% (*P*< 0.05). Animal numbers are listed in the figure legends and were based on the FDA guidelines for pharmacokinetic studies recommending at least 3–4 animals per time point. (Ref attached) GraphPad Prism software v6.0 (GraphPad Software Inc. San Diego, CA) was used for all statistical analyses.

## Results

### Intranasal administration increased dantrolene peak concentration and duration in the brain

We have compared the dantrolene pharmacokinetics both in plasma and brain after oral and intranasal administration.

**Fig 1:**
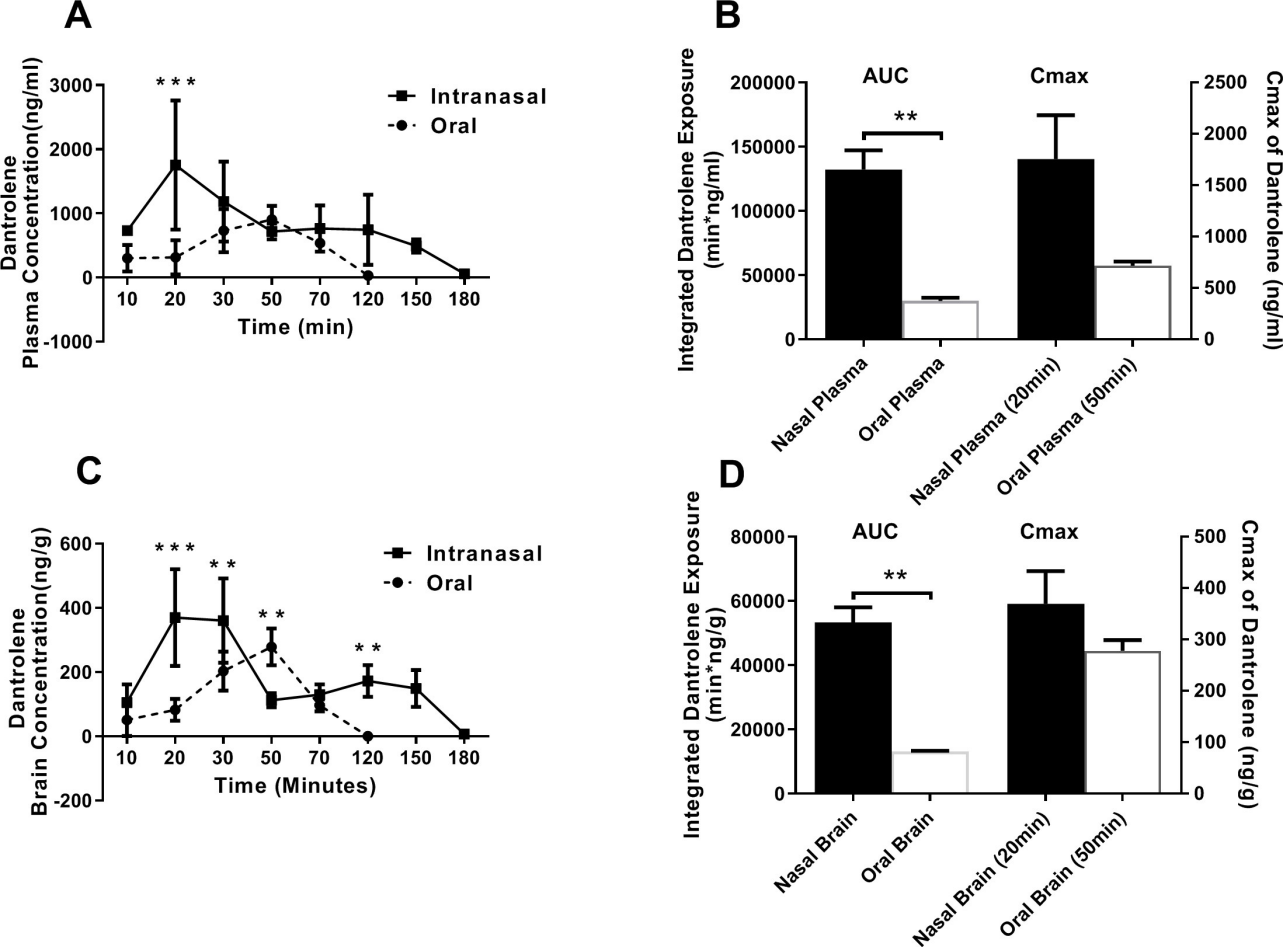
Pharmacokinetic analysis of dantrolene in plasma and brain after oral and intranasal administration. **A.** The peak dantrolene plasma concentration (Cmax) occurred at 20 minutes after intranasal administration (5mg/kg) and at 50 minutes after oral administration (5mg/kg). ***p = 0.0000089 compared to oral administration determined with multiple t-tests using the Sidak-Holm method, alpha = 0.05%. Intranasal time points, 10, 30, 150,180 min, n = 5; 20 min, n = 8; 50–120 min, n = 4; oral time points, 10–120 min; n = 5. **B**. Comparison of integrated dantrolene exposure (areas under the curves of panel A) (left) and Cmax (right) in plasma after intranasal and oral administration of dantrolene, **p = 0.0079 with the nonparametric unpaired Mann-Whitney test (two-tailed). Nasal Plasma (20 min), n = 8; Oral Plasma, (50 min), n = 4; **C.** The brain concentration of dantrolene after intranasal administration (5mg/kg) was greater than after oral administration at most time points. The Cmax occurred at 20 minutes after intranasal administration and 50 minutes after oral administration, respectively. ***p = 0.00000012, **p = 0.0035 (30 min), **p = 0.0037 (50 min), **p = 0.0027 (120 min), compared to contrast group (intranasal vs. oral) and determined with multiple t-tests using the Sidak-Holm method, alpha = 0.05%. Intranasal time points, 10, 30, 150,180 min, n = 5; 20 min, n = 8; 50–120 min, n = 4; oral time points, 10–120 min; n = 5. **D**. Comparison of integrated dantrolene exposure in brain tissue after intranasal and oral administration of dantrolene (areas under the curves of panel C) and Cmax in brain (right) after intranasal and oral administration of dantrolene, **p = 0.0079 with the nonparametric unpaired Mann-Whitney test (two-tailed). Nasal Brain and Oral Brain, n = 5; Nasal Brain (20 min), n = 8; Oral Brain (50) min, n = 5. All data are expressed as Mean ± 95%CI.

With intranasal dantrolene administration, the concentration of dantrolene in the plasma (Fig 1A) and brain (Fig 1C) was significantly greater at 20 min (p<0.001 and p<0.001, respectively) compared to the oral administration. After 20 min, the intranasal administration dantrolene plasma levels gradually decreased (Fig 1A), though remained elevated through 150 min and still detectable at 180 min. The brain dantrolene concentrations (Fig 1C) remained significantly elevated at 30 (p = 0.0035) and 120 min (p = 0.0027) compared to the oral approach, and remained elevated out to 150 min, and then decreasing, though detectable, at 180 min. The dantrolene concentrations in the plasma with the oral approach gradually increased to a peak at 50 min, followed by a decline to zero values at 120 min (Fig 1A). Similarly, the brain dantrolene concentrations with oral administration were significantly elevated at 50 min (p = 0.0037), followed by a more rapid decline (Fig 1C). Accordingly, the integrated dantrolene exposure in brain but not plasma was significantly higher after intranasal than oral administration (Fig 1B and 1D, left panels). The peak dantrolene concentration (Cmax) in both the plasma and brain after intranasal administration was higher than those after the oral route, though not statistically significant (Fig 1B and 1D, right panels).

### Intranasal delivery increased dantrolene passage across the blood brain barrier (BBB)

**Fig 2:**
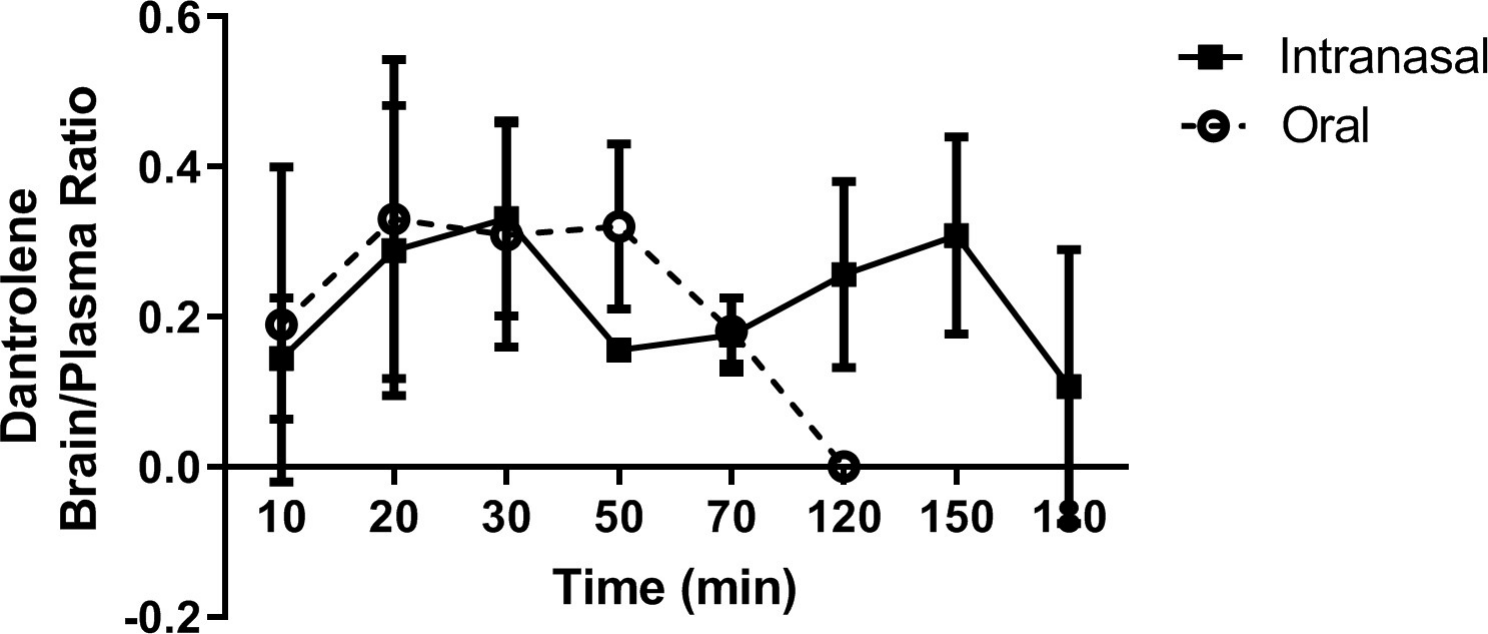
The dantrolene concentrations in the brain over time after intranasal vs oral administration. There were no significant differences in the dantrolene brain/plasma ratios between intranasal and oral administration. The oral brain/plasma ratio fell to zero while the intranasal dantrolene brain/plasma ratio was sustained through 180 min. Data are expressed as Mean ± 95% CI, significance determined by multiple t-tests using the Holm-Sidak method with alpha = 5.00%. %. Intranasal time points, 10, 30, 150,180 min, n = 5; 20 min, n = 8; 50–120 min, n = 4; oral time points, 10–120 min; n = 5.

To examine whether the intranasal dantrolene increased the passage of dantrolene across the BBB, we compared the brain-to-plasma dantrolene concentration ratios, a commonly used indicator for drug penetration into the brain. There were no significant differences in the brain/plasma dantrolene concentration ratios. At 120 minutes, the plasma and brain dantrolene concentrations reached zero after oral administration (Fig 1A and 1C; Fig 2), while with intranasal administration, the dantrolene brain/plasma concentration ratios maintained relatively high levels up to 180 min. (Fig 2).

### Chronic use of intranasal dantrolene did not impair olfaction or motor function

**Fig 3:**
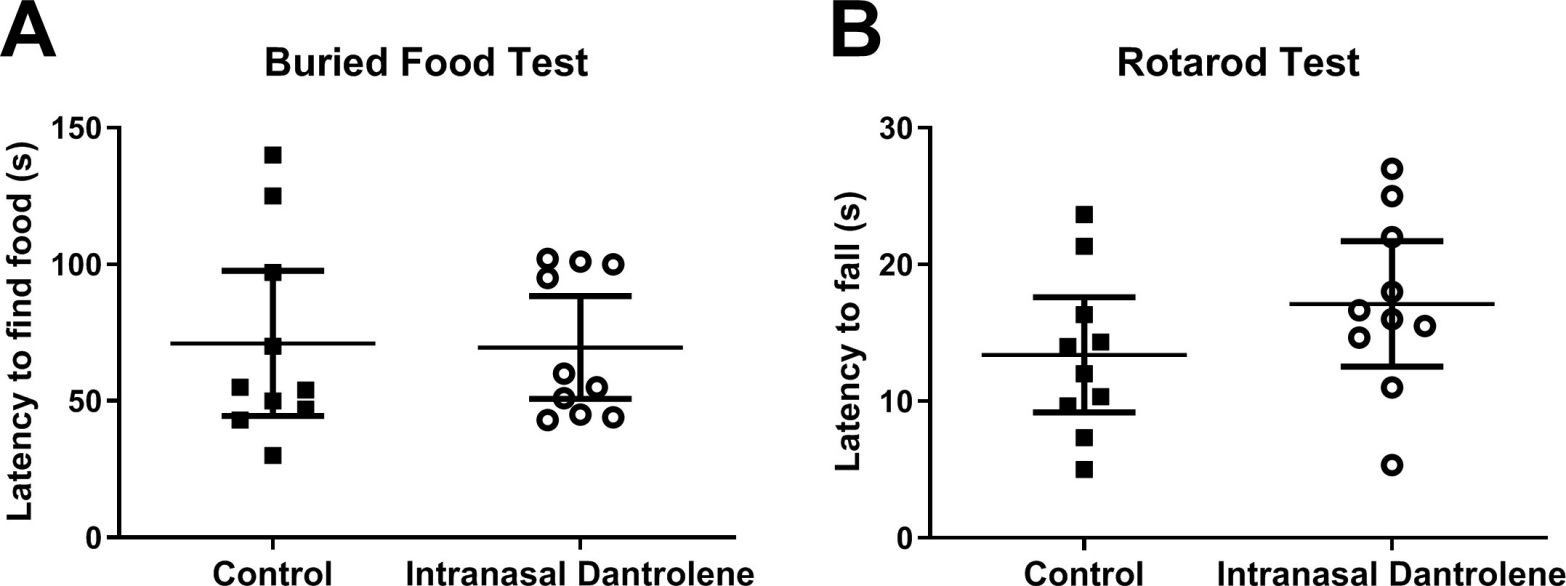
Long-term intranasal administration of dantrolene did not affect olfaction or motor function. **A.** After 3 weeks of intranasal administration of dantrolene (5mg/kg, 3 times/wk) or vehicle control, olfaction was measured by the time in seconds (s) necessary for the animal to retrieve the buried food with its front paws. **B.** After 4 months of intranasal administration of dantrolene (5mg/kg, 3 times/wk), motor function was determined by the length of time manually recorded for latency to find food (**A**) but automatically recorded for the animal spent on the rotarod (**B**). No significant differences were detected in olfaction or motor function. Data are expressed as the Mean ± 95% CI, analyzed with the nonparametric unpaired Mann-Whitney test, n = 10 for all groups.

To examine the possible adverse side effects chronic intranasal administration of dantrolene on olfaction, we performed the food buried test. Intranasal dantrolene had no significant change on olfaction in mice after 3 weeks of intranasal administration at 5 mg/kg, three times a week. (Fig 3A).

As dantrolene is a muscle relaxant, we examined motor coordination using the rotarod test. Intranasal dantrolene treatment (5mg/kg, 3 times/week) for four months did not affect the muscle strength significantly compared to control (Fig 3B)

### P-gp/BCRP inhibition did not increase dantrolene concentrations in the brain

**Fig 4:**
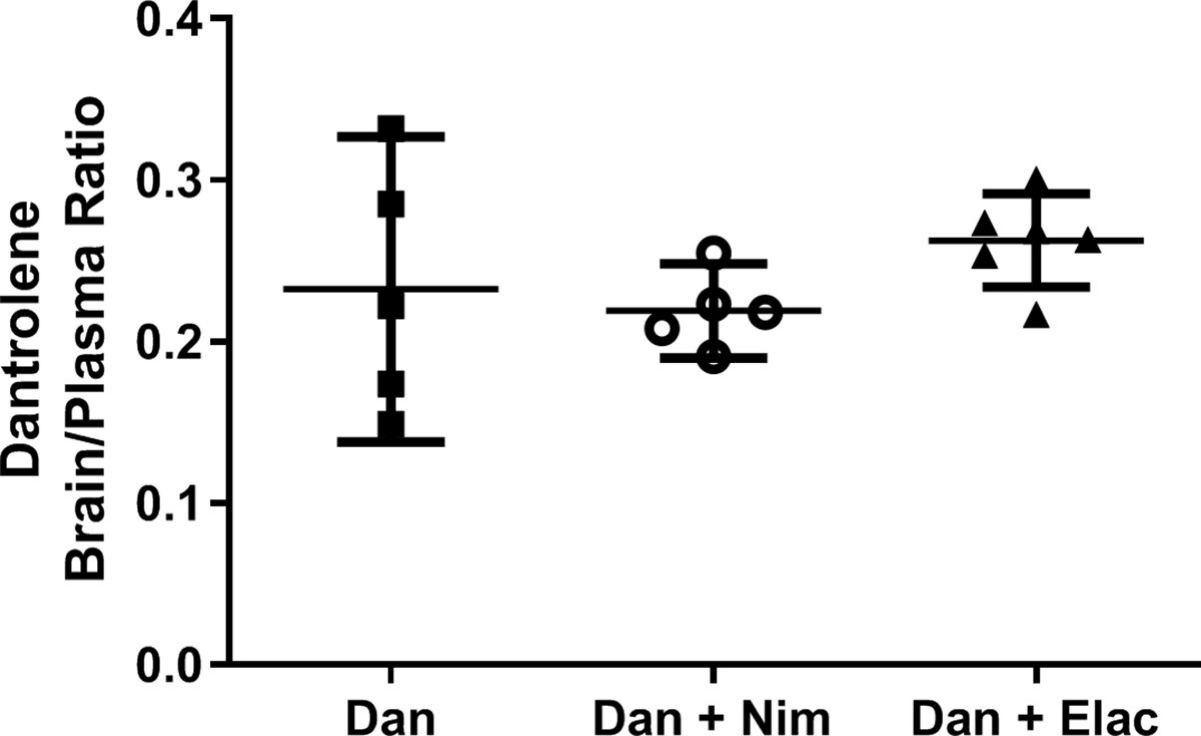
Blood brain barrier (BBB) inhibitors, nimodipine and elacridar, had no effect on dantrolene passage. After 20 minutes of internasal administration of dantrolene (5mg/kg), in the presence or absence of BBB pump inhibitors (P-gp/BCRP), nimodipine (Nim, 2mg/kg) or elacridar (Elac, 10mg/kg) dissolved in the same vehicle as Ryanodex, the dantrolene brain/plasma ratios were determined as a measure of dantrolene passage across the BBB. No significant differences were detected with either inhibitor compared to dantrolene alone. The data are expressed as the Mean ± 95% CI, n = 5 (Dan, Dan + Nim), n = 6 (Dan + Elac, and analyzed with the Kruskall-Wallis non-parametric ANOVA with Dunn’s multiple correction test.

Previous studies indicated that the BBB transport protein, P-glycoprotein breast cancer resistance (P-gp/BCRP), on the cerebral vascular endothelium, may function as a pump that drives dantrolene out of the brain, contributing to the limited dantrolene penetration into the brain [24]. We therefore examined whether the P-gp/BCRP inhibitors, nimodipine or elacridar, would increase dantrolene brain concentrations. Neither nimodipine nor elacridar significantly increased dantrolene brain/dantrolene plasma concentration ratios (Fig 4).

## Discussion

To our knowledge, this is the first study investigating dantrolene brain pharmacokinetics after intranasal administration. We found that intranasal administration of dantrolene, when compared with oral administration, significantly increased dantrolene peak concentrations and duration in the brain, without obvious side effects on olfaction or motor function. Previous studies have found dantrolene to be neuroprotective in various cell and animal models of neurodegenerative diseases [25]. For example, in a spinocerebellar ataxia type 3 animal model, brain dantrolene concentration at 30 minutes after oral administration was as 67 nM and this concentration was found to be neuroprotective [26]. The results of this study demonstrate much higher brain concentrations (479 nM at 150 min, Fig 1C) with intranasal delivery and similar levels in the blood as with the oral approach, which allows for therapeutic concentrations in the brain without systemic side effects. Furthermore, the duration of dantrolene in the brain was much longer after intranasal administration than the oral approach, making the overall drug exposure in the brain significantly increased. The intranasal administration of dantrolene may provide an important new therapeutic approach for clinical studies on neurodegenerative diseases. It should be noted that parallel to its higher brain concentrations, the plasma concentrations at most times after intranasal administration were also higher than oral approach, although the brain/plasma concentration ratios were similar between two administration methods. More translational studies are needed to compare the side effects and toxicity between intranasal administration of dantrolene and commonly used oral or intravenous approaches.

Although intranasal administration has been used to increase drug penetration into the brain by enhancing the passage of drugs across the BBB, the brain-to-plasma ratios in this study did not find an increased passage of dantrolene across the BBB, compared to the oral approach, during first 120 min. After 120 minutes, the oral administration ratios fell to zero while the intranasal ratios were sustained, due to the sustained brain concentrations. The mechanism for how intranasal dantrolene increased the brain concentrations and durations, compared to oral approach, is unclear. We have proposed the following mechanisms to explain this phenomenon. 1) Rich nasal vascular structure improved dantrolene absorption, making its concentration in both the plasma and brain high. 2) The intranasal delivery using the Ryanodex formula enhances dantrolene adherence to the nasal mucosal and sustained drug absorption. 3) Drug metabolism by the liver is reduced using the nasal approach. It should be noted that plasma concentrations after intranasal administration are higher than those after oral administration in parallel to its higher brain concentrations. Future studies need to investigate the optimal intranasal concentration to achieve the highest brain concentrations with the lowest plasma concentrations.

Previous studies have indicated that there is limited penetration of dantrolene into the brain after oral or intravenous administration [15]. One of the proposed mechanisms is that P-gp/BCRP proteins, on the cerebrovascular endothelium, pump dantrolene out of the brain [24]. However, at least in our study, this does not appear to be the case. Nimodipine, an L-type of voltage dependent calcium channel (VDCC) blocker, which inhibits calcium influx into neurons, has been demonstrated to be neuroprotective in various neurodegenerative diseases including AD [5, 20]. In combination with dantrolene, which inhibits excessive Ca^2+^ release from the ER, may theoretically provide synergistic neuroprotection and requires further study. Although nimodipine can inhibit P-gp/BCRP [27], our results do not support that nimodipine increased dantrolene brain concentrations.

In other studies, oral dantrolene administration for up to 8 months did not cause significant muscle weakness or other chronic toxicity [20, 23]. Patients with multiple sclerosis are prescribed dantrolene for muscle spasms, at doses up to 100mg, four times a day. This study further demonstrates that the intranasal administration of dantrolene for three weeks did not affect olfaction, nor motor function after four months of treatment. These results suggest that chronic nasal administration of dantrolene is relatively safe, making its long-term use for the treatment of AD feasible, although further studies are needed.

Dantrolene is an FDA approved drug to treat malignant hyperthermia, neuroleptic malignant syndrome and muscle spasm etc., with tolerable side effects and toxicity [28] [29] Clinical use of dantrolene up to now primarily targeted to inhibition of type 1 ryanodine receptors in the skeletal muscles. Although dantrolene has been demonstrated to be neuroprotective in different animal models of various neurodegenerative diseases, few clinical studies investigate its potential neuroprotection in patients with various neurodegenerative diseases, considering its limitation to penetrate into CNS [5, 15] and its dose-dependent feature of neuroprotection [30] The relatively increased brain concentrations at most times and prolonged durations after intranasal administration compared to the oral approach demonstrated in this study has suggested a new approach to optimize the dantrolene neuroprotection for future treatment of various neurodegenerative disease, especially for those with chronic development, such as Alzheimer’s and Huntington disease. Supporting this hypothesis, our recent study [31] suggested that chronic administration of intranasal dantrolene provided better therapeutic effects to ameliorate cognitive dysfunction, with similar tolerable side effects compared to intraparietal administration of dantrolene at same doses. In addition, intranasal administration of neuroprotective drugs is also considered a convenient approach for those patients with difficulty to take medicine orally or intravenously, such as in children or in non-cooperated patients (e.g. dementia). For example, intranasal pyrrolidine dithiocarbamate has been conveniently and successfully used for neuroprotection after brain hypoxia–ischemia in in neonatal rats. [32] Pending the further translational studies on efficacy and safety of intranasal dantrolene in animal models of various neurodegenerative diseases, it is hoped that intranasal dantrolene can be eventually studied in patients with various neurodegenerative diseases, especially in Alzheimer’s disease.

The study is limited in the following aspects: 1) The accuracy of the dantrolene dose given to the mice could be affected by the lack of general anesthesia during intranasal drug administration. 2) Incomplete perfusion of the cerebral vasculature during euthanasia may lead to inconsistent dantrolene brain concentration measurements. 3) Since we measured the dantrolene concentration in the brain tissue, we did not measure the dantrolene concentrations in the CSF in these mice. 4) The vehicle for dantrolene is the ryanodex formula which contains several agents that may affect passage of dantrolene across the BBB, and we did not set up another vehicle control to rule out this possibility. 5). We did not include a group with intravenous administration of dantrolene for comparison, which will be proposed in future studies.

In summary, intranasal administration of dantrolene significantly increased the brain peak concentration and duration and overall exposure, without obvious side effects on smell or motor function, providing a new potential approach for augmenting dantrolene neuroprotection in various CNS neurodegenerative diseases.

## Supporting information

S1 File. Dataset (ZIP)

## References

[1] Larach MG, Brandom BW, Allen GC, Gronert GA, Lehman EB. Cardiac arrests and deaths associated with malignant hyperthermia in north america from 1987 to 2006: a report from the north american malignant hyperthermia registry of the malignant hyperthermia association of the United States. Anesthesiology. 2008;108(4):603–11. Epub 2008/03/26. 10.1097/ALN.0b013e318167aee2 . 18362591

[2] Britt BA, Kalow W. Malignant hyperthermia: a statistical review. Can Anaesth Soc J. 1970;17(4):293–315. Epub 1970/07/01. 10.1007/bf03004694 . 4246871

[3] Nakamura-Maruyama E, Miyamoto O, Okabe N, Himi N, Feng L, Narita K, et al Ryanodine receptors contribute to the induction of ischemic tolerance. Brain Res Bull. 2016;122:45–53. Epub 2016/03/02. 10.1016/j.brainresbull.2016.02.018 . 26930163

[4] Kocogullari CU, Emmiler M, Cemek M, Sahin O, Aslan A, Ayva E, et al Can dantrolene protect spinal cord against ischemia/reperfusion injury? An experimental study. Thorac Cardiovasc Surg. 2008;56(7):406–11. Epub 2008/09/24. 10.1055/s-2008-1038731 . 18810698

[5] Chen X, Wu J, Lvovskaya S, Herndon E, Supnet C, Bezprozvanny I. Dantrolene is neuroprotective in Huntington's disease transgenic mouse model. Mol Neurodegener. 2011;6:81 Epub 2011/11/29. 10.1186/1750-1326-6-81 22118545 PMC3235068

[6] Staats KA, Van Rillaer M, Scheveneels W, Verbesselt R, Van Damme P, Robberecht W, et al Dantrolene Is Neuroprotective in Vitro, but Does Not Affect Survival in Sod1(G93a) Mice. Neuroscience. 2012;220:26–31. 10.1016/j.neuroscience.2012.06.050 WOS:000307802100004. 22750242

[7] Rosado IR, Lavor MS, Alves EG, Fukushima FB, Oliveira KM, Silva CM, et al Effects of methylprednisolone, dantrolene, and their combination on experimental spinal cord injury. Int J Clin Exp Pathol. 2014;7(8):4617–26. Epub 2014/09/10. 25197334 PMC4152024

[8] Keshavarz M, Fotouhi M, Rasti A. Dantrolene: A selective ryanodine receptor antagonist, protects against pentylenetetrazole-induced seizure in mice. Acta Medica Iranica. 2016;54(9):555–61. 27832686

[9] Celes MR, Malvestio LM, Suadicani SO, Prado CM, Figueiredo MJ, Campos EC, et al Disruption of calcium homeostasis in cardiomyocytes underlies cardiac structural and functional changes in severe sepsis. PLoS One. 2013;8(7):e68809 10.1371/journal.pone.0068809 23935889 PMC3720843

[10] Chakroborty S, Briggs C, Miller MB, Goussakov I, Schneider C, Kim J, et al Stabilizing ER Ca2+ channel function as an early preventative strategy for Alzheimer’s disease. PloS one. 2012;7(12):e52056 10.1371/journal.pone.0052056 23284867 PMC3528716

[11] Peng J, Liang G, Inan S, Wu Z, Joseph D, Meng Q, et al Early and chronic treatment with dantrolene blocked later learning and memory deficits in older Alzheimer's triple transgenic mice. Alzheimers Dement. 2011;7(4):e67.

[12] Oules B, Del Prete D, Greco B, Zhang X, Lauritzen I, Sevalle J, et al Ryanodine receptor blockade reduces amyloid-beta load and memory impairments in Tg2576 mouse model of Alzheimer disease. J Neurosci. 2012;32(34):11820–34. Epub 2012/08/24. 10.1523/JNEUROSCI.0875-12.2012 22915123 PMC3458216

[13] Chakroborty S, Briggs C, Miller MB, Goussakov I, Schneider C, Kim J, et al Stabilizing ER Ca(2+) Channel Function as an Early Preventative Strategy for Alzheimer's Disease. PLoS One. 2012;7(12):e52056 10.1371/journal.pone.0052056 23284867 PMC3528716

[14] Meyler WJ, Bakker H, Kok JJ, Agoston S, Wesseling H. The effect of dantrolene sodium in relation to blood levels in spastic patients after prolonged administration. J Neurol Neurosurg Psychiatry 1981;44(4):334–9. Epub 1981/04/01. 10.1136/jnnp.44.4.334 7241161 PMC490957

[15] Wuis EW, Rijntjes NV, Van der Kleijn E. Whole-body autoradiography of 14C-dantrolene in the marmoset monkey. BMC Pharmacol Toxicol. 1989;64(1):156–8. Epub 1989/01/01. 10.1111/j.1600-0773.1989.tb00621.x . 2502774

[16] Lochhead JJ, Thorne RG. Intranasal delivery of biologics to the central nervous system. Adv Drug Deliv Rev. 2012;64(7):614–28. 10.1016/j.addr.2011.11.002 . 22119441

[17] Renner DB, Svitak AL, Gallus NJ, Ericson ME, Frey WH 2nd, Hanson LR. Intranasal delivery of insulin via the olfactory nerve pathway. J Pharm Pharmacol. 2012;64(12):1709–14. 10.1111/j.2042-7158.2012.01555.x . 23146033

[18] Thorne R, Hanson L, Ross T, Tung D, Frey W. Delivery of interferon-β to the monkey nervous system following intranasal administration. Neuroscience. 2008;152(3):785–97. 10.1016/j.neuroscience.2008.01.013 18304744

[19] Dhuria SV, Hanson LR, Frey WH 2nd. Intranasal delivery to the central nervous system: mechanisms and experimental considerations. J Pharm Sci. 2010;99(4):1654–73. Epub 2009/10/31. 10.1002/jps.21924 . 19877171

[20] Wu Z, Yang B, Liu C, Liang G, Eckenhoff MF, Liu W, et al Long-term dantrolene treatment reduced intraneuronal amyloid in aged Alzheimer triple transgenic mice. Alzheimer Dis Assoc Disord. 2015;29(3):184–91. 10.1097/WAD.0000000000000075 25650693 PMC4543576

[21] Kodaira H, Kusuhara H, Fujita T, Ushiki J, Fuse E, Sugiyama Y. Quantitative evaluation of the impact of active efflux by p-glycoprotein and breast cancer resistance protein at the blood-brain barrier on the predictability of the unbound concentrations of drugs in the brain using cerebrospinal fluid concentration as a surrogate. J Pharmacol Exp Ther. 2011;339(3):935–44. Epub 2011/09/22. 10.1124/jpet.111.180398 . 21934030

[22] Yang M, Crawley JN. Simple behavioral assessment of mouse olfaction. Curr Protoc Neurosci. 2009;Chapter 8:Unit 8.24. Epub 2009/07/04. 10.1002/0471142301.ns0824s48 19575474 PMC2753229

[23] Peng J, Liang G, Inan S, Wu Z, Joseph DJ, Meng Q, et al Dantrolene ameliorates cognitive decline and neuropathology in Alzheimer triple transgenic mice. Neurosci Lett. 2012;516(2):274–9. Epub 2012/04/21. 10.1016/j.neulet.2012.04.008 22516463 PMC3351794

[24] Fuchs H, Kishimoto W, Gansser D, Tanswell P, Ishiguro N. Brain penetration of WEB 2086 (Apafant) and dantrolene in Mdr1a (P-glycoprotein) and Bcrp knockout rats. Drug Metab Dispos 2014;42(10):1761–5. Epub 2014/07/24. 10.1124/dmd.114.058545 . 25053619

[25] Wei H, Perry DC. Dantrolene is cytoprotective in two models of neuronal cell death. J Neurochem. 1996;67(6):2390–8. 10.1046/j.1471-4159.1996.67062390.x 8931471

[26] Chen X, Tang TS, Tu H, Nelson O, Pook M, Hammer R, et al Deranged calcium signaling and neurodegeneration in spinocerebellar ataxia type 3. J Neurosci. 2008;28(48):12713–24. 10.1523/JNEUROSCI.3909-08.2008 19036964 PMC2663415

[27] Zhang Y, Gupta A, Wang H, Zhou L, Vethanayagam RR, Unadkat JD, et al BCRP transports dipyridamole and is inhibited by calcium channel blockers. Pharm Res. 2005;22(12):2023–34. Epub 2005/10/26. 10.1007/s11095-005-8384-4 . 16247709

[28] Ward A, Chaffman MO, Sorkin EM. Dantrolene. A review of its pharmacodynamic and pharmacokinetic properties and therapeutic use in malignant hyperthermia, the neuroleptic malignant syndrome and an update of its use in muscle spasticity. Drugs. 1986;32(2):130–68. Epub 1986/08/01. 10.2165/00003495-198632020-00003 . 3527659

[29] Krause T, Gerbershagen MU, Fiege M, Weisshorn R, Wappler F. Dantrolene—a review of its pharmacology, therapeutic use and new developments. Anaesthesia. 2004;59(4):364–73. Epub 2004/03/17. 10.1111/j.1365-2044.2004.03658.x . 15023108

[30] Inan S, Wei H. The cytoprotective effects of dantrolene: a ryanodine receptor antagonist. Anesth Analg. 2010;111(6):1400–10. Epub 2010/09/24. 10.1213/ANE.0b013e3181f7181c . 20861418 PMC9382853

[31] Shi Y, Zhang L, Gao X, Zhang J, Ben-Abou M, Meng Q, et al INTRANASAL DANTROLENE AS A DISEASE-MODIFYING DRUG IN ALZHEIMER 5XFAD MICE. Alzheimers Dement. 2019;15(7):P597. 10.3233/JAD-200227 PMC7505009 32623395

[32] Wang Z, Zhao H, Peng S, Zuo Z. Intranasal pyrrolidine dithiocarbamate decreases brain inflammatory mediators and provides neuroprotection after brain hypoxia-ischemia in neonatal rats. Exp Neurol. 2013;249:74–82. Epub 2013/09/03. 10.1016/j.expneurol.2013.08.006 23994718 PMC3833659

